# Oligodendrocytes are susceptible to Zika virus infection in a mouse model of perinatal exposure: Implications for CNS complications

**DOI:** 10.1002/glia.24010

**Published:** 2021-05-04

**Authors:** Verena Schultz, Jennifer A. Barrie, Claire L. Donald, Colin L. Crawford, Margaret Mullin, Thomas J. Anderson, Tom Solomon, Susan C. Barnett, Christopher Linington, Alain Kohl, Hugh J. Willison, Julia M. Edgar

**Affiliations:** ^1^ College of Medical, Veterinary, and Life Sciences Institute of Infection, Immunity and Inflammation Glasgow UK; ^2^ MRC‐University of Glasgow Centre for Virus Research Glasgow UK; ^3^ School of Veterinary Medicine, College of Medical Veterinary and Life Sciences Glasgow; ^4^ Institute of Infection, Veterinary and Ecological Sciences, University of Liverpool Liverpool UK

**Keywords:** apoptosis, inflammasome, neurodevelopmental delay, white matter

## Abstract

Some children with proven intrauterine Zika virus (ZIKV) infection who were born asymptomatic subsequently manifested neurodevelopmental delays, pointing to impairment of development perinatally and postnatally. To model this, we infected postnatal day (P) 5–6 (equivalent to the perinatal period in humans) susceptible mice with a mammalian cell‐propagated ZIKV clinical isolate from the Brazilian outbreak in 2015. All infected mice appeared normal up to 4 days post‐intraperitoneal inoculation (dpi), but rapidly developed severe clinical signs at 5–6 dpi. All nervous tissue examined at 5/6 dpi appeared grossly normal. However, anti‐ZIKV positive cells were observed in the optic nerve, brain, and spinal cord; predominantly in white matter. Co‐labeling with cell type specific markers demonstrated oligodendrocytes and astrocytes support productive infection. Rarely, ZIKV positive neurons were observed. In spinal cord white matter, which we examined in detail, apoptotic cells were evident; the density of oligodendrocytes was significantly reduced; and there was localized microglial reactivity including expression of the NLRP3 inflammasome. Together, our observations demonstrate that a clinically relevant ZIKV isolate can directly impact oligodendrocytes. As primary oligodendrocyte cell death can lead later to secondary autoimmune demyelination, our observations may help explain neurodevelopmental delays in infants appearing asymptomatic at birth and commend lifetime surveillance.

## MAIN POINTS

- In a mouse model of perinatal Zika virus infection, oligodendrocytes are susceptible.
- NLRP3 expression is upregulated.
- Myelin defects may explain neurodevelopmental delays in congenitally infected infants who were asymptomatic at birth.

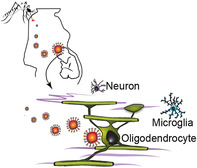

## 1 INTRODUCTION

Zika virus (ZIKV; *Flaviviridae*, *Flavivirus*) infection can cause developmental malformations in children born to infected mothers; so‐called congenital Zika syndrome (CZS; Araujo, Silva, & Araujo, 2016). Microcephaly is the best‐known neurological abnormality associated with CZS (Driggers et al., 2016; Li et al., 2016; Mlakar et al., 2016; Rasmussen, Jamieson, Honein, & Petersen, 2016; Tang et al., 2016). However, a vast range of other neurological signs and symptoms have been reported including visual and hearing deficits, seizure activity, hypertonicity, spasticity, hyperreflexia, contractures, dysphagia, nystagmus, and feeding difficulties (Walker et al., 2019). In addition, recent reports demonstrate that children with congenital ZIKV infection who appeared asymptomatic at birth, subsequently manifest progressive neurodevelopmental delays (Familiar et al., 2020; Mulkey et al., 2020; Peçanha et al., 2020; Pimentel et al., 2021).

The CNS is susceptible to congenital infection through vertical transmission during the entire gestational period (Brasil et al., 2016); however, infection in the earlier weeks of the embryonic phase generally results in more severe malformations (Chimelli et al., 2017 and reviewed in Saad et al., 2018). This likely reflects the susceptibility of neural progenitor cells (NPCs), resulting in changes in gene expression, impaired proliferation and migration, and apoptotic cell death (Cugola et al., 2016; Garcez et al., 2017; Li et al., 2016; Souza et al., 2016; Tang et al., 2016). Nonetheless, CNS abnormalities have been reported following infection as late as 39 weeks of gestation, demonstrating that later developmental processes are also susceptible (Brasil et al., 2016).

In developing humans, neurogenesis largely occurs during the first semester (Kostović, Sedmak, & Judaš, 2019) and following this, precursors contribute to gliogenesis during which astrocytes and oligodendrocytes are produced (DeAzevedo et al., 2003; Rash et al., 2019). Myelin production by oligodendrocytes, is well‐underway in the third trimester (Kostović et al., 2019; Poduslo & Jang, 1984) and is largely complete by 2 years of postnatal life (Kinney & Volpe, 2018). Although it is not yet known if ZIKV infection directly affects developmental myelination in humans, other human and/or murine neurotropic viruses including Semliki Forest virus (SFV), Theiler's virus, cytomegalovirus (CMV), and John Cunningham virus (JCV), infect and/or injure oligodendroglia leading to dysmyelination or demyelination (Dal Canto & Lipton, 1975; Hayden et al., 2020; Rorke et al., 1979; Tan & Koralnik, 2010).

To determine if oligodendrocytes are susceptible to ZIKV during myelination in vivo, we infected P5/6 mice with ZIKV, some days after CNS myelination commences at P1. We mainly used mice lacking the type I interferon receptor (*Ifnar1* knockout mice), which recapitulate aspects of human ZIKV infections and disease (Miner & Diamond, 2017), to approximate ZIKV antagonism of the type I interferon (IFN) response in humans (Serman & Gack, 2019). Immunohistochemistry and cell quantification revealed that oligodendrocytes were particularly vulnerable. The functional outcome could not be determined, as mice had to be euthanized due to rapid development of severe clinical disease. However, in other contexts, oligodendrocyte death is followed after some delay, by loss of compact myelin (Pohl et al., 2011; Traka et al., 2010). Further, our data may explain neurodevelopmental delays in some congenitally infected infants and warn of susceptibility to later autoimmune mediated demyelination (Traka, Podojil, McCarthy, Miller, & Popko, 2016).

## 2 METHODS AND MATERIALS

### 2.1 Mice

*Ifnar1* knockout (KO; type I interferon receptor deficient) and wild type (WT) mice of both sexes, on a 129S7/SvEvBrdBkl‐Hprtb‐m2 background (B&K Universal) were maintained in Tecniplast 1284L Blue line IVC cages, in a 12 hr light/dark cycle and provided with sterile food and water ad libitum. All animal studies were approved by the Ethical Committee of the University of Glasgow and licensed by the UK Home Office (Project Licence numbers PPL P78DD624O and P9722FD8E). Genomic DNA was extracted from ear biopsies using a modified protocol (Truett et al., 2000). Briefly, ear notches were heated to 95°C for 90 min in 50 mM NaOH. Following neutralization with 10% v/v 1 M Tris pH 5, the resultant solution was vortexed to release DNA and 2 μl was used for PCR as described previously (Cumberworth et al., 2017).

### 2.2 Cell lines and Zika virus

As indicated in Section 3, we used both low passage ZIKV, isolated from a patient in Brazil with febrile illness, ZIKV*/H*. *sapiens/*Brazil/PE243/2015 (GenBank accession number KX197192; abbreviated to ZIKV PE243) and the prototypic African ZIKV isolate, MR766/1947/Uganda (abbreviated to ZIKV MR766). The origin and history of ZIKV PE243 have been previously described (Donald et al., 2016). ZIKV MR766 was obtained from BEI Resources, NIAID, NIH: Genomic RNA from ZIKV Virus, MR766, NR‐50085 (Zmurko et al., 2018). The viral stocks were generated using either the Vero E6 cell line (ATCC, CCL‐81), A549/BVDV‐NPro cell line (kindly gifted by R.E. Randall, University of St Andrews, UK) or the *Aedes albopictus*‐derived C6/36 cell line. Stocks were titred using the A549/BVDV‐NPro cell line as described previously (Donald et al., 2016). The Vero E6 cell line was grown at 37°C with 5% CO_2_ in DMEM (Life Technologies) supplemented with 10% fetal bovine serum (FBS, Life Technologies), and penicillin–streptomycin (final concentration 100 units/ml and 100 μg/ml, respectively, Life Technologies). The A549/BVDV‐NPro cell line was grown under the same conditions but with the addition of puromycin (2 μg/ml, Invivogen). The C6/36 cell line was grown at 28°C with no additional CO_2_ in L‐15+ Glutamax (Life Technologies) supplemented with 10% FBS, penicillin–streptomycin (final concentration 100 units/ml and 100 μg/ml, respectively) and 10% Tryptose Phosphate Broth (TPB, Life Technologies).

### 2.3 Infection of mice with Zika virus

At P5/6, pups of both sexes were removed from the dams and administered an intraperitoneal (ip) injection with 7.5 × 10^4^ to 7.5 × 10^5^ (Table S1) plaque forming units (PFU) of ZIKV per animal, or with an equivalent volume of vehicle only (cell culture media), using a 1 ml syringe or 5 μl Hamilton syringe. Each litter received both virus and vehicle, providing littermate controls for virally infected animals. Pups were randomly assigned to each of the two groups. Six *Ifnar1* knockout litters and two wild type litters were used (Table S1).

### 2.4 Examination and assessment of mice

Mice were examined twice daily following inoculation. Their behavior was assessed visually by one or other of two experienced observers while the pups were in the cage together with the dam, or out of the cage without the dam. Videos of the mice were examined by TJA (veterinary neurologist) who provided clinical descriptions. Pups were humanely killed at timepoints indicated in Table 1, usually being when clinical signs were observed, or as age‐matched controls to sick animals. Most pups were immersion fixed in approximately 100 ml 8% paraformaldehyde (PFA) in phosphate buffered saline (PBS) for 2–3 days before dissection. To facilitate impregnation with fixative, pups were decapitated, and a vertical incision was made in the skin on the back and front of the body and the skin pared away from the muscles. Two litters of *Ifnar1* knockout mice were perfusion fixed in 8% paraformaldehyde or 4% paraformaldehyde and 5% glutaraldehyde (the latter for electron microscopy) at 4 dpi, as described (Edgar, Smith, & Duncan, 2020).

**TABLE 1:** Clinical signs in wild type (WT) and *Ifnar1* ^*−/−*^ mice after infection with mammalian cell‐propagated ZIKV PE243 or ZIKV MR766 (i.p.)

Viral isolate, day of assessment and numbers	WT		*Ifnar1* ^*−/−*^	
	PE243 (at 9 dpi, when 1 mouse showed clinical signs; *n* = 4)	MR766 (at 6–7 dpi, when 2 mice showed clinical signs; *n* = 4)	PE243 (at 5–6 dpi; *n* = 13)	MR766 (at 4–5 dpi; *n* = 3)
Found dead	0/4	0/4	6/13	1/3
Urinary retention	1/4	2/4	6^a^/7	2/2
Piloerection	1/4	2/4	6^a^/7	2/2
Hunched posture	1/4	2/4	6^a^/7	2/2
Weakness/paralysis of hindlimbs	0/4	1/4	4^a^/7	2/2
Failure to correct itself when on back	0/4	0/4	2/7	2/2
Unresponsive to external stimuli	0/4	0/4	1/7	1/2
Dehydration	1/4	2/4	6^a^/7	2/2
Cold to touch	1/4	2/4	6^a^/7	2/2

^a^ One animal was infected with insect cell‐propagated ZIKV PE243 and appeared clinically similar to the remainder infected with mammalian cell‐propagated ZIKV PE243. dpi, days post‐infection; i.p., intraperitoneal. Thirteen *Ifnar1* ^*−/−*^ mice killed at 4 dpi are not shown in the table as they appeared clinically normal at that time.

### 2.5 Tissue preparation and immunohistochemical staining

The brain, spinal cord, and optic nerve were dissected and placed for further fixation in 4% PFA in PBS overnight. The tissue was then transferred into 20% sucrose in PBS until it sank (usually overnight), embedded in Tissue‐Tek OCT medium (Sakura Europe), and rapidly frozen in liquid nitrogen chilled isopentane. Optic nerves were frozen flat between discs of frozen OCT (approximately −23°C). Transverse sections of forebrain and cervical and lumbar spinal cord, and longitudinal sections of optic nerves, were cut at 12‐μm thickness and collected on plus charged slides (VWR or Waldemar Knittel) at 96‐μm intervals. Sections were stored in a −20°C freezer until required. Slides were allowed to reach room temperature before they were removed from their storage box, then washed in PBS to remove OCT. Tissues were permeabilized using −20°C ethanol, 10 min, −20°C Methanol, 10 min (for CD3), or 0.5% TritonX in PBS, 10 min (for CD68), then sections were washed in PBS, blocked in 10% normal goat serum (NGS) in PBS or 10% horse serum and 1% BSA in PBS (HS‐BSA) for 1 hr. Primary antibodies (below) were applied in 10% NGS or HS‐BSA overnight at 4°C. After thorough washing in PBS, secondary antibodies (below), diluted 1:1,000 were applied for 1 hr at room temperature. Sections were washed three times in PBS and once in distilled water, then mounted with CitiFLUOR (Electron Microscopy Science, Hatfield, Pennsylvania) or Mowiol mounting medium [4.2% glycerol (w/v), 0.4% Mowiol 4–88 (w/v) (Calbiochem, San Diego, California), 2.1% 0.2 M Tris pH 8.5 (v/v)] with DAPI (1 ng/ml).

Primary and secondary antibodies were: mouse IgG1 anti‐ZIKV Envelope protein (clone 0302156 Aalto Bio; 1 in 500); mouse IgG2a anti‐OLIG2 (MABN50, clone 211F1.1, Millipore; 1 in 200); mouse IgG2b anti‐APC (clone CC1, NB600‐1021 Novus biologicals; 1:200); rabbit IgG anti‐NeuN (ABN78 Millipore; 1 in 750), rabbit IgG anti‐S100B (PA5 78161, Invitrogen; 1 in 400), rabbit IgG anti‐GFAP (Z0334, Dako; 1 in 1,000); rabbit IgG Iba1 antibody (019‐19741, Wako/Alpha laboratories; 1 in 500), rat IgG anti‐MBP (MCA409S AbD, Serotec; 1 in 500); mouse IgG1 SMI31 anti‐phosphorylated heavy (H) and medium (M) chain neurofilament (NF) (801601, Biolegend; 1 in 1,500); rat IgG anti‐CD3 (MCA1477, BioRad; 1 in 200); rat IgG anti‐CD68 (MCA1957GA); rabbit IgG anti‐cleaved caspase 3 (Ab49822, Abcam, 1 in 200), rabbit IgG anti‐NLRP3 (NBP1‐77080, Novus Biologicals, 1 in 100); goat anti‐mouse IgG1 (A21121 and A21125); goat anti‐rat IgG (A11006 and A11007); goat anti‐rabbit IgG (A11008 and A11037); goat anti‐mouse IgG2a (A21135); or goat anti‐mouse IgG2b (A21145), all secondaries, conjugated with Alexa 488, Alexa 594, or Alexa 647 (Invitrogen; all 1 in 1,000).

### 2.6 Microscopy and cell quantification

To quantify cells across spinal cord transverse sections, images of immunostained nervous tissue were captured, as illustrated in Figure 5, at ×40 magnification using an Olympus IX70 microscope with standard epifluorescence optics and Image Pro Plus 6 software. Cell counts were made in areas of interest (AOI) of 44,835 μm^2^ (cell‐type specific marker and anti‐ZIKV envelope protein) or 5,400 μm^2^ (DAPI +ve nuclei). The experimenter was blinded to the conditions and the field of view was selected in the blue channel (DAPI) to avoid biased selection. One to four sections per spinal cord, at least 50‐μm apart, were analyzed (2–10 images/section). To quantify the proportion of ZIKV +ve cells that were CC1 +ve, or cleaved caspase 3 +ve cells that were ZIKV and/or CC1 +ve, images were captured in four channels at sites where ZIKV +ve cells were present, at ×20 magnification, using a Zeiss Axio Imager M.2 with an AxioCam MRm and Zen 2012 blue edition (version 1.1.2.0). Up to three sections, 50‐μm apart, were imaged of both cervical and lumbar spinal cord, from two animals (1–3 AOI per section). Representative images for illustration were obtained using the Olympus IX70 microscope as above; an Olympus BX51 fluorescence microscope and Ocular software (QImaging); a Zeiss Axio Imager M.2 with an AxioCam MRm and Zen 2012 blue edition (version 1.1.2.0); or a Zeiss LSM 880 inverted confocal microscope and Zen Black software.

### 2.7 Quantification of myelin and axon volumes

Fluorescence images of myelin basic protein (MBP; labeled in green) and phosphorylated H‐neurofilament and M‐neurofilament (NF; labeled in red) were captured of the cervical and lumbar cord white matter (input images). These were transformed in Cell Profiler (Jones et al., 2008) to binary images (output images) and the total pixels per AOI, red pixels per AOI and green pixels per AOI were quantified to provide a readout of the relative volumes occupied by myelin or axons; being red or green pixels as a percentage of all pixels, as described previously (Bijland et al., 2019).

### 2.8 Resin sections and electron microscopy

At 4 dpi, mice were rapidly perfusion fixed in 4% paraformaldehyde, 5% glutaraldehyde in cacodylate buffer and tissue was processed, stained, and imaged as described (Edgar et al., 2020).

### 2.9 Statistical analysis

Analyses were performed using Graphpad Prism 8.3.0 software (GraphPad Software Inc., San Diego, California). The *p* values ≤.05 were considered significant. An unpaired two‐tailed Student's *t*‐test was used to compare cell densities between mock‐infected (*n* = 3 independent animals) and PE243 ZIKV‐infected (*n* = 3 independent animals) *Ifnar1* knockout animals at 5 days post‐infection with ZIKV.

## 3 RESULTS

### 3.1 Neonatally infected mice develop rapidly progressing disease

Experimental studies on ZIKV infection in mice have used a variety of mouse strains, ages of infection, viral strains, and viral propagation protocols. Here we infected P5/6 (equivalent to the perinatal period in humans, with respect to myelination) *Ifnar1* knockout mice, in which ZIKV replicates efficiently (Lazear et al., 2016), with clinically relevant ZIKV PE243. We used mammalian cell‐propagated virus to emulate viral replication in somatic cells following mosquito bite. For comparison with other studies, we also examined a small number of identically housed wild type mice on the same A129 background, and a small number of animals of both genotypes infected with mammalian cell‐propagated ZIKV MR766, a mouse brain‐passaged isolate (Dick, 1952) from a sentinel monkey in the Zika Forest (Table S1).

**FIGURE 1:**
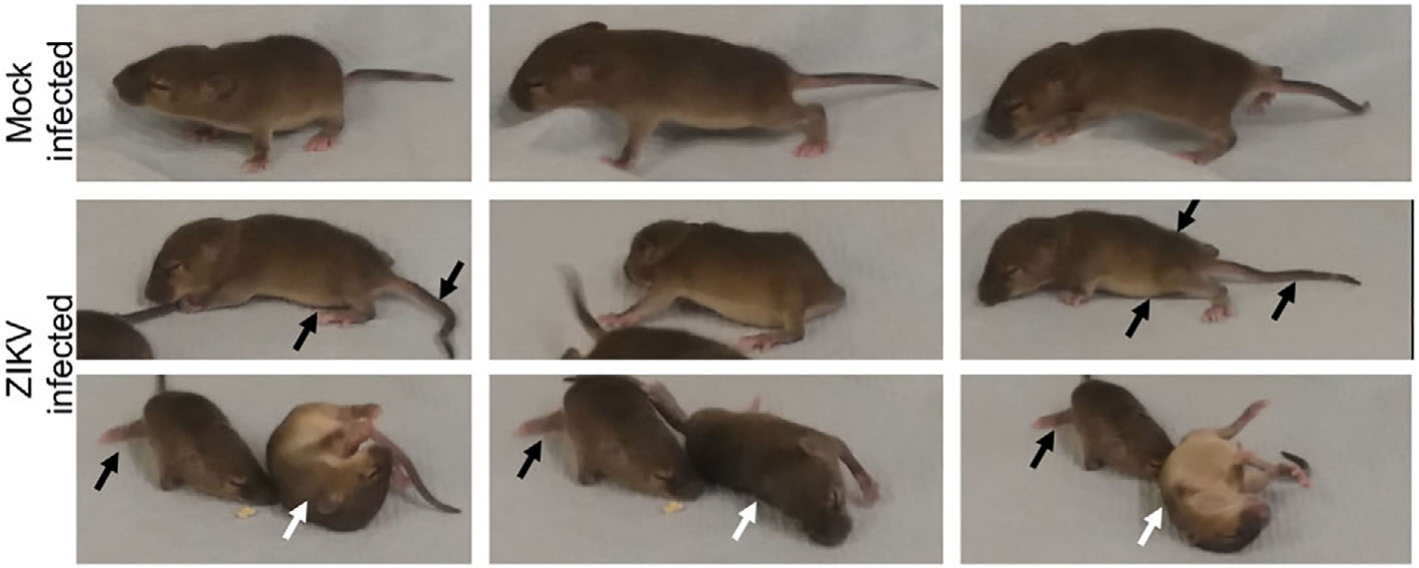
*Ifnar1* knockout mice developed clinical signs 5–6 dpi ZIKV PE243. Sequential frames from videos of one mock‐infected animal and its three ZIKV PE243 infected littermates. Clinical signs included failure to bear weight, weakness or paralysis of hindlimbs, flacid tail and failure to right. Black arrows in the lower images indicate abnormal positioning of the back, abdomen, tail and hindlimbs. White arrows indicate failure of righting [Color figure can be viewed at wileyonlinelibrary.com]

*Ifnar1* knockout mice infected with ZIKV PE243 developed clinical signs and died or had to be euthanized at 5 or 6 dpi. Signs included wide stance and failure to bear body weight, flaccid tail, urinary retention, weakness or paralysis of hindlimbs, or failure of righting (Figure 1). The progression of clinical signs occurred rapidly, where some mice showing no, or only very mild signs in late afternoon, were found moribund or dead the following morning. Over and above the clinical neurological phenotype, an overwhelming viral infection and systemic antiviral inflammatory response may have contributed to the moribund state and early death in some mice, but this was not investigated further.

In contrast, only one of four wild type mice infected with ZIKV PE243 displayed signs of disease, occurring at 9 dpi. Three of three *Ifnar1* knockout mice (two litters) infected with ZIKV MR766 became severely clinically compromised and were found dead or had to be euthanized at 4–5 dpi. Two of four wild type mice (two independent litters) infected with the same isolate showed clinical signs at 6 or 7 dpi and were euthanized. No mock‐infected littermate of either genotype developed clinical signs (12 *Ifnar1* ko and two wild type mice). Clinical observations in infected mice are summarized in Table 1. Numbers of mice, strains, viral isolates, and PFUs are summarized in Table S1.

In summary, all infected *Ifnar1* knockout mice maintained beyond 4 dpi, succumbed to disease, independent of the viral isolate. In wild type mice, the response to infection was variable, irrespective of the viral isolate.

### 3.2 Zika virus positive cells are present as clusters in the CNS

**FIGURE 2:**
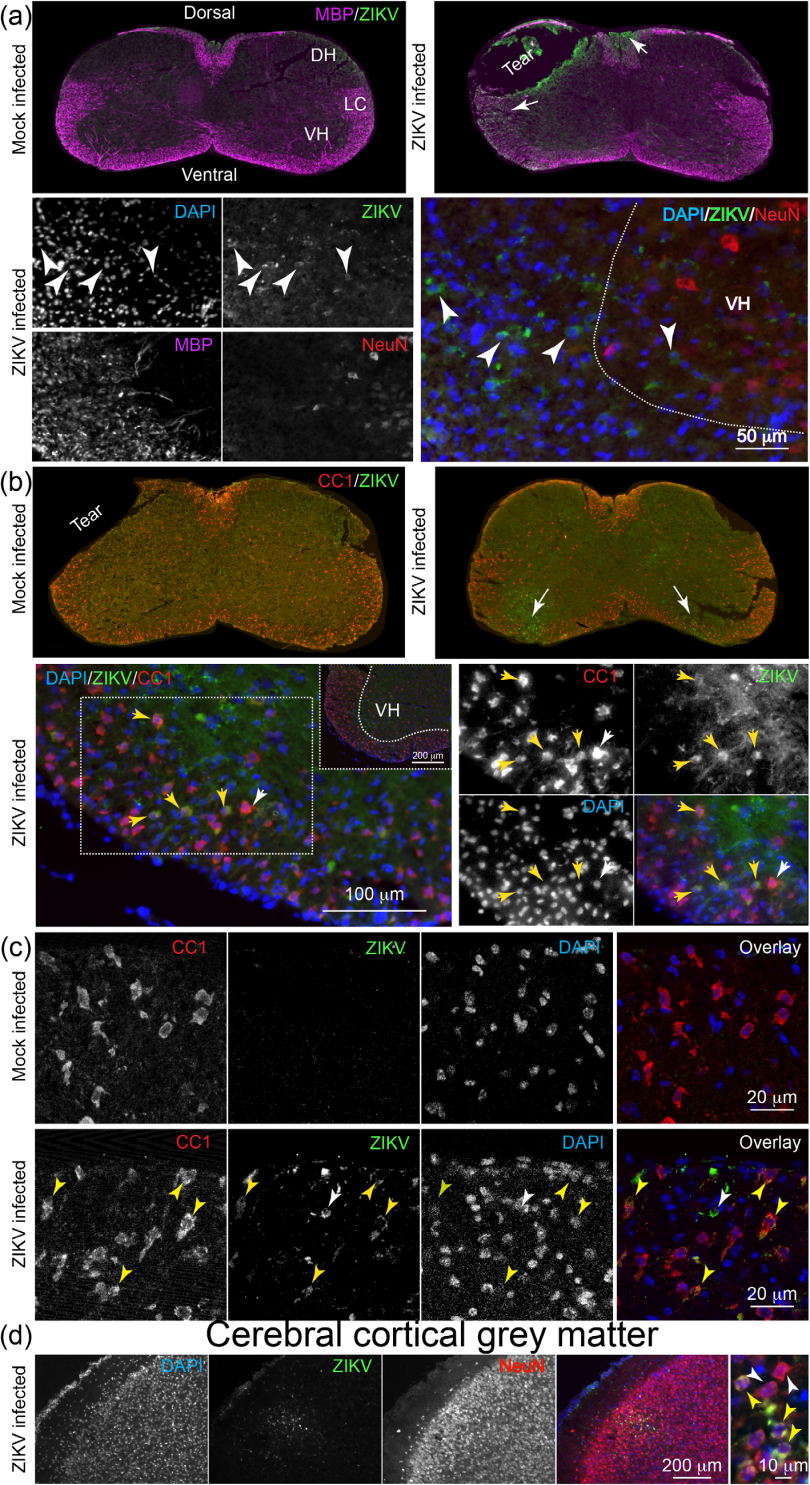
ZIKV positive cells in the CNS white matter at 4 dpi. Fluorescence micrographs of immunostained CNS. (a) Overviews of cervical spinal cord sections of mock‐infected and ZIKV‐infected mice, stained with antibody to ZIKV and myelin basic protein (MBP). Clusters of ZIKV +ve cells are seen in the dorsal and lateral columns (arrows) in the infected animal. In the higher magnification view (lower panel) of the border between the ventral horn and ventrolateral columns of another infected animal, small numbers of ZIKV +ve cells can be seen (white arrowheads), mainly in the white matter. NeuN +ve neurons in the gray matter are not co‐labeled with anti‐ZIKV. In the overlay, MBP has been removed for clarity. (b) Overviews of cervical spinal cord sections of mock‐infected and ZIKV‐infected mice, stained with anti‐ZIKV and antibody clone CC1, which recognizes mature oligodendrocytes. ZIKV +ve cells can be seen in the ventral white matter on both sides (arrows) and in the ventral horn on left side of the image of the infected animal. In the higher magnification view (lower panel) of the ventrolateral white matter of an infected animal, small numbers of ZIKV +ve cells are positive also for CC1 (yellow arrowheads). The white arrowhead indicates an adjacent CC1 +ve cell that is not anti‐ZIKV +ve. (c) Single confocal planes of the ventrolateral white matter of a mock‐infected and ZIKV‐infected animal, showing colocalization of anti‐ZIKV and antibody clone CC1 (yellow arrowheads) in the infected animal. The white arrowhead indicates a ZIKV +ve cell that is not co‐labeled with antibody CC1. (d) ZIKV +ve cells were observed less frequently in the gray matter; here the cerebral cortex. A region of infected cells, in cortical layers II/III, is shown at low magnification in relation to NeuN +ve neurons. The high magnification image on the right shows NeunN +ve cells that are ZIKV +ve (yellow arrows) and NeuN +ve cells that are ZIKV −ve (white arrows). DH, dorsal horn; VH, ventral horn; LC, lateral column [Color figure can be viewed at wileyonlinelibrary.com]

Following infection at P5/6 with ZIKV PE243 (19 mice; six litters), seven infected and six mock‐infected *Ifnar1* knockout littermates (two independent litters) were culled at 4 dpi without overt clinical signs. Using immunohistochemistry on CNS tissue of three infected and three control mice, we found small clusters of ZIKV +ve cells in all CNS areas examined, being cervical spinal cord, cerebral cortex (Figure 2), lumbar spinal cord, and cerebellum (Figure S1). ZIKV +ve cells were observed most frequently in white matter regions including the ventrolateral columns (Figure 2a–c) and dorsal columns of the spinal cord, the cerebellum, and the brain stem. Less frequently, we observed ZIKV +ve cells in gray matter (Figure 2d). Mock‐infected littermates appeared negative for anti‐ZIKV (Figure 2a,b).

### 3.3 The CNS appears grossly normal in clinically affected ZIKV‐infected animals

**FIGURE 3:**
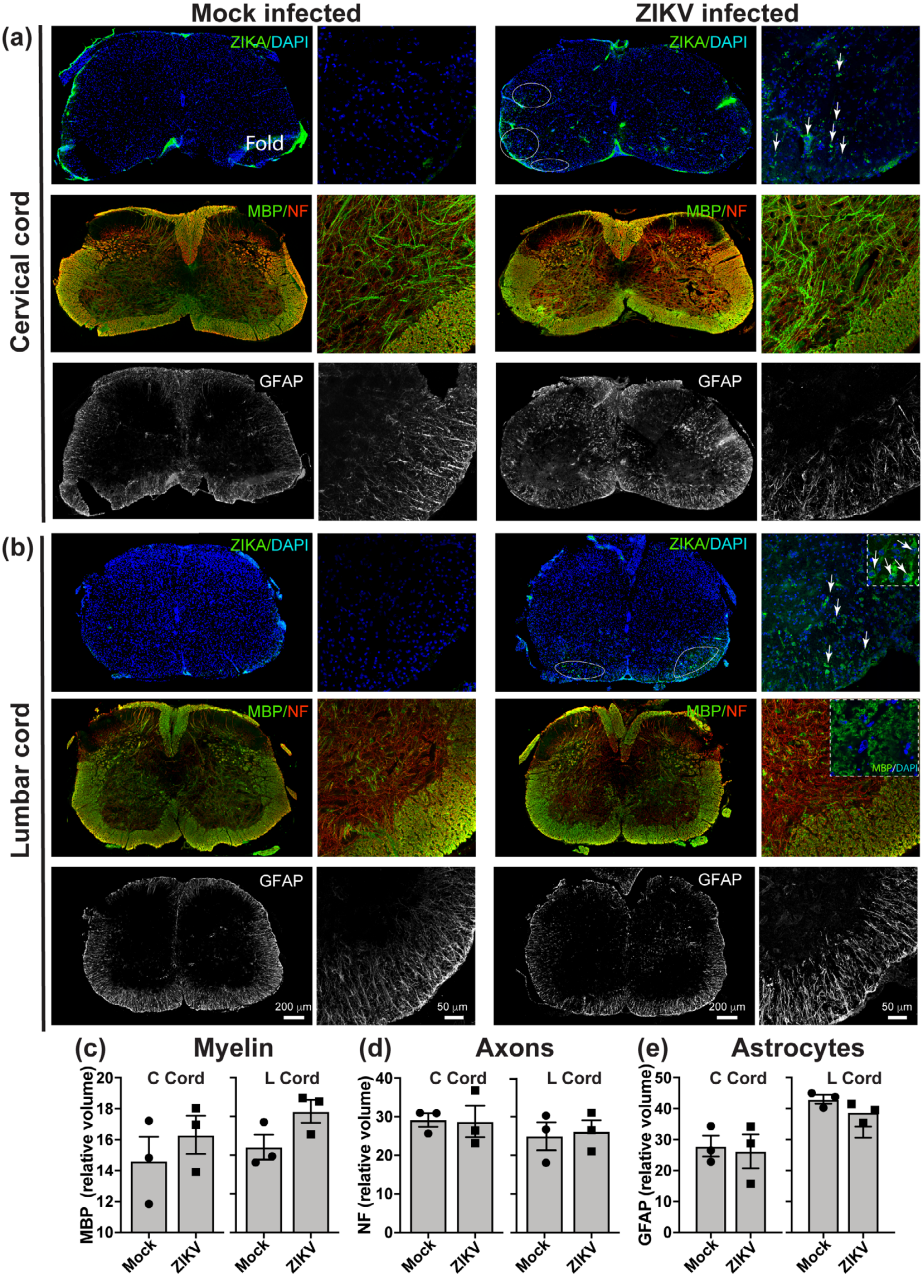
The spinal cord appears grossly normal in clinically affected mice. Immunofluorescence images of transverse sections of (a) cervical and (b) lumbar spinal cord of mock and ZIKV PE243 infected *Ifnar1* knockout mice at 5 or 6 dpi, when infected mice displayed severe clinical signs. Spinal cord overviews are widefield epifluorescence images and higher magnification images are confocal maximum intensity projections (MIPs) of the ventrolateral columns and adjacent dorsal horn gray matter. ZIKV +ve cells were present, particularly in white matter, indicating productive infection. Grossly, myelin (MBP staining) and axons (NF staining) appeared intact at this time point. GFAP staining of astrocytes, appeared similar in mock‐infected and ZIKV‐infected animals. The insert in b, upper right panel, shows a higher magnification view of ZIKV +ve cells. The inset in b, middle right panel, shows a single Z plane to illustrate MBP +ve myelin rings. (c–e) Quantification of myelin (MBP), axons (NF), and astrocytes (GFAP) staining in white matter revealed no significant differences in the volume of myelin, axons, or astrocytes in ZIKV‐infected mice compared with mock‐infected controls. In c–e, Y‐axes values are indicated on the graph on the left. Each data point represents the average value from one independent animal. Bars indicate mean ± *SEM* [Color figure can be viewed at wileyonlinelibrary.com]

To determine whether CNS changes might contribute to clinical signs, we used immunohistochemical staining of nervous tissue of clinically affected mice (5 or 6 dpi) to examine localization and frequency of infected cells and overall tissue integrity. As at 4 dpi, ZIKV +ve cells were distributed heterogeneously throughout the nervous system, particularly as clusters in white matter of the spinal cord (Figure 3a,b and Figure S1) and cerebellum (Figure S1). Due to these observations and because clinical signs (urinary retention and hindlimb weakness) suggested spinal cord involvement, we examined the cervical and lumbar cord in more detail. Grossly, the overall appearance of the spinal cord at 5/6 dpi, including axons, myelin and astrocytes was comparable to that of mock‐infected animals (Figure 3a,b). Furthermore, quantification of immunohistochemical staining revealed similar relative volumes of myelin (MBP staining), axons (neurofilament staining), and astrocytes (GFAP staining), compared with mock‐infected controls (Figure 3c–e).

### 3.4 Zika virus‐infection leads to cell death

**FIGURE 4:**
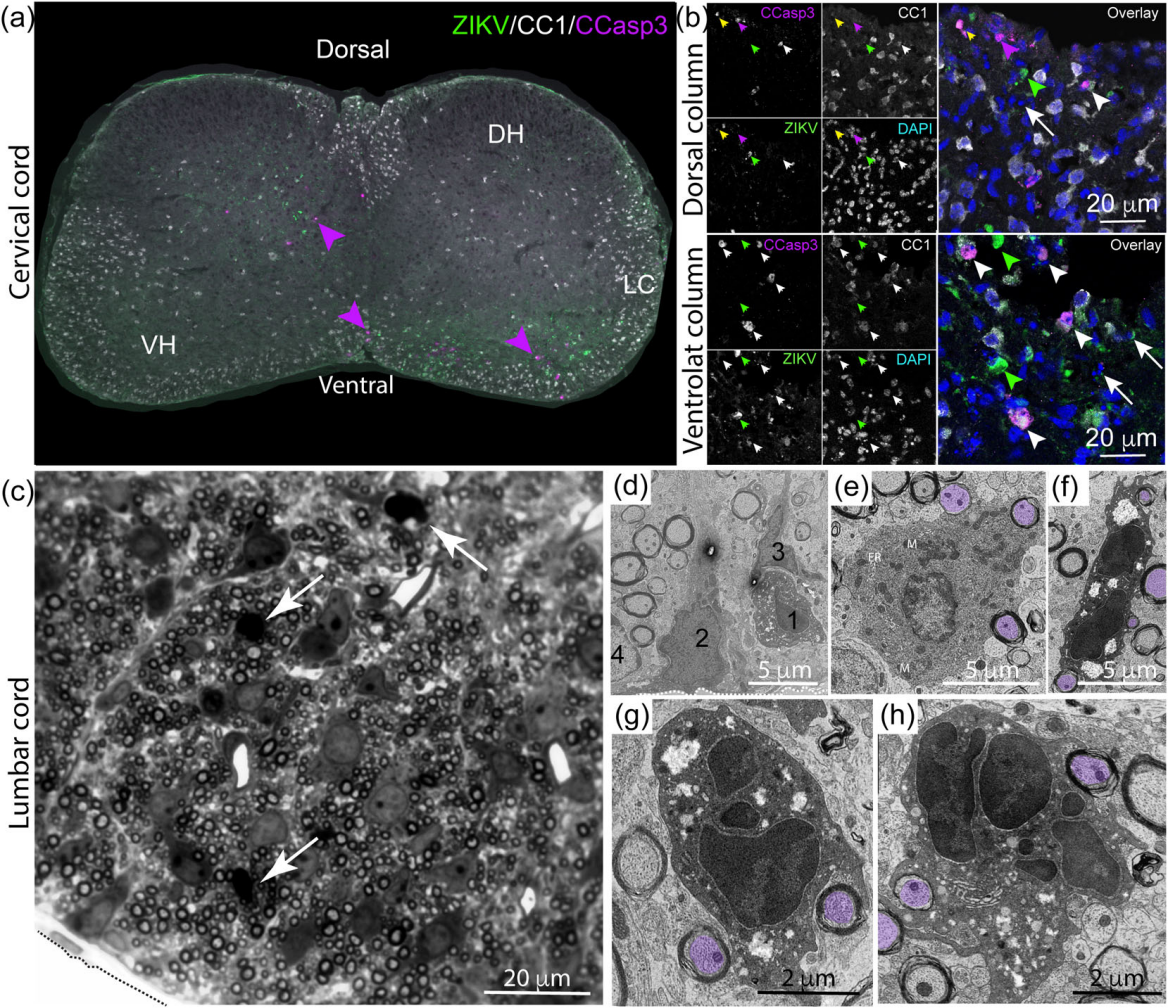
Zika virus infection induces apoptosis. (a) Immunofluorescence micrograph of spinal cord of an *Ifnar1* knockout mouse at 4 dpi stained with anti‐ZIKV, anti‐CC1 (to label oligodendrocytes) and anti‐cleaved caspase 3, demonstrates apoptotic cells in proximity to ZIKV +ve cells (magenta arrowheads). DH: dorsal horn; VH: ventral horn; LC: lateral columns. (b) Single Z plane confocal images from dorsal horn or ventrolateral columns of two more animals. Cells positive for cleaved caspase 3 +ve alone (magenta arrowheads) were observed. Some cleaved caspase 3 +ve cells co‐labeled with antibody CC1 (white arrowheads), which labels mature oligodendrocytes, and some cells were co‐labeled with anti‐cleaved caspase 3, CC1, and anti‐ZIKV (yellow arrow). Some ZIKV +ve cells did not co‐label with either cleaved caspase 3 or CC1 (green arrowheads). Pyknotic or karryhexic nuclei (white arrows) were also observed in close proximity to ZIKV +ve cells. (c) Resin section of the ventrolateral columns (tissue edge delineated by dashed line) demonstrates that pyknotic nuclei (white arrows) are interspersed with healthy‐appearing nuclei. (d) Electron micrograph of white matter (tissue edge delineated by dashed line) confirms that a dying cell (1) is interspersed with healthy appearing cells (2–4). (e) Normal‐appearing oligodendrocyte, wrapping axons (purple overlays), has a dark cytoplasm and contains many mitochondria (M) and endoplasmic reticulum (ER). (f–h) Dying oligodendrocytes, still wrapping axons (purple overlays), have a densely labeled cell nucleus and very dark cytoplasm which appears vacuolated [Color figure can be viewed at wileyonlinelibrary.com]

Although the CNS appeared grossly normal despite the severe clinical signs, we next asked whether ZIKV‐infection caused localized cell death. At 4 dpi, when mice appeared clinically unremarkable, we found cleaved caspase 3 +ve cells localized to regions containing ZIKV +ve cells (magenta arrowheads Figure 4a). To determine if the dying cells were oligodendrocytes, which are highly abundant in white matter, we co‐labeled tissue with antibody clone CC1 [a marker that labels oligodendrocytes but not oligodendrocyte progenitor cells (OPCs)], anticleaved caspase 3 and anti‐ZIKV (Figure 4b, showing single z plane confocal images of dorsal and ventrolateral spinal cord white matter). We found cells labeled only with anti‐cleaved caspase 3 (Figure 4 b, magenta arrowhead); some also with anti‐ZIKV [52 (±27.94 *SD*) % of cleaved caspase 3 +ve cells; 63 cells observed in sections of cervical and lumbar cord from 2 animals; Figure 4b, green arrowhead], rare cells positive only for CC1 and cleaved caspase 3 (Figure 4b, white arrowheads); and some positive for all three markers (Figure 4b, yellow arrowhead [16 (±4.82 *SD*) % of cleaved caspase 3+ ve cells, 63 cells observed in sections of cervical and lumbar cord from two animals]. Some ZIKV +ve cells (green arrowheads) and pyknotic or karryhexic nuclei (white arrows) were not co‐labeled with either anticleaved caspase 3 or antibody CC1. In resin sections obtained from a separate litter, we observed pyknotic nuclei in spinal cord white matter of infected mice (Figure 4c, white arrows) interspersed amongst healthy‐appearing cell nuclei. Electron microscopy confirmed the presence of dying cells (Figure 4d, cell 1) interspersed amongst healthy‐appearing cells (Figure 4d, cells 2–4), including healthy‐appearing (Figure 4e) and dying (Figure 4f–h) oligodendrocytes. In summary, dying cells, including early myelinating oligodendrocytes, were observed in white matter of ZIKV PE243 infected mice.

### 3.5 Oligodendrocytes are particularly vulnerable following Zika virus infection

**FIGURE 5:**
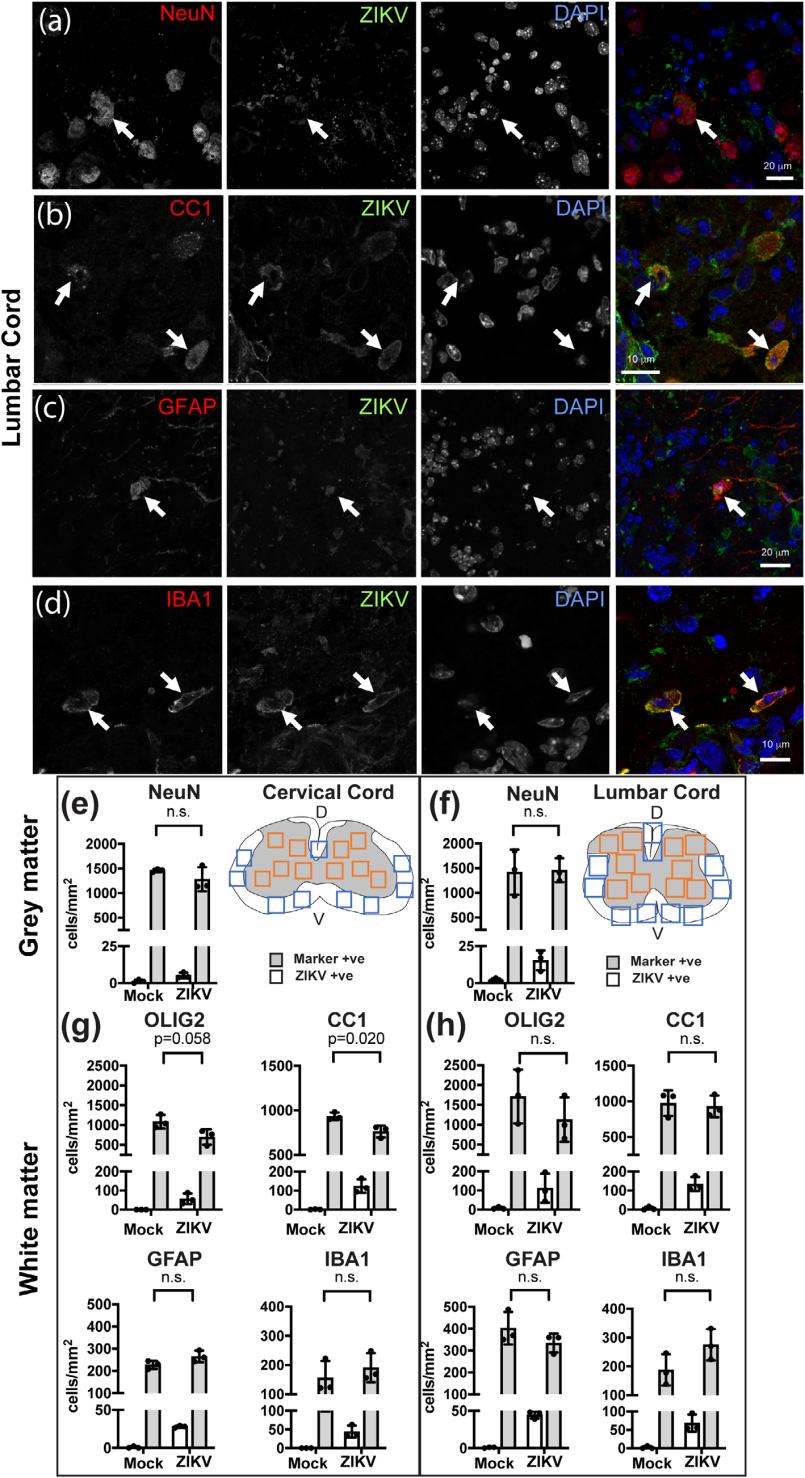
ZIKV infected all major cell types, but only oligodendroglial densities were reduced. (a–d) Representative single Z plane confocal images of ZIKV‐infected cells at 5 dpi (NeuN +ve neurons, CC1 +ve oligodendrocytes; GFAP +ve astrocyte; IBA1 +ve microglia). (e, f) Schematics of cervical and lumbar spinal cord show approximately where images were captured for quantification of neurons in gray matter (orange boxes) and glia in white matter (blue boxes). Dorsal (D) and ventral (V) aspects are indicated. (e–h) All investigated cell types (densities represented by gray bars) were susceptible to infection (white bars). Densities of CC1 +ve oligodendrocytes were significantly reduced in ZIKV‐infected animals compared with mock‐infected controls, in cervical spinal cord. A very small number of cells in the mock‐infected controls appeared positive for ZIKV, likely reflecting autofluorescence due to lipofuscin. Graphs indicate mean ± *SEM*; *p* ≤ .05 was considered significant [Color figure can be viewed at wileyonlinelibrary.com]

As shown in Figures 2, 3, 4, most ZIKV +ve cells appeared to be in white matter. We confirmed this by quantifying the proportion of ZIKV +ve cells in cervical and lumbar cord white or gray matter (Table 2). Next, we used co‐labeling with anti‐ZIKV and cell type specific markers to determine which cell types support productive ZIKV infection. We found all major neural cell types, being neurons (Figure 5a), oligodendrocytes (Figure 5b), astrocytes (Figure 5c), and microglia/macrophages (Figure 5d) could be productively infected. We did not examine OPCs because the fixation required to inactivate ZIKV was not compatible with antibody staining for NG2, a classical marker for OPCs.

**TABLE 2:** Percentage ZIKV positive cells in white and gray matter of the spinal cord

Region	White matter	Gray matter
	Percentage ZIKV positive cells	
Cervical cord	6.6 (±1.1 *SEM*)	1.8 (±0.5 *SEM*)
Lumbar cord	9.8 (±1.0 *SEM*)	2.1 (±0.3 *SEM*)

To determine if cell death observed at 4 dpi (Figure 4) led to a significant reduction in cell densities, we quantified individual cell types in cervical and lumbar spinal cord sections in animals at 5/6 dpi. The density of NeuN +ve neurons was similar in mock‐infected and ZIKV‐infected animals (gray bars, Figure 5e,f). In cervical cord white matter, densities of OLIG2 +ve oligodendroglia (oligodendrocytes and OPCs) tended to be reduced in infected mice compared with mock‐infected controls, although the difference was not significant (Figure 5g; *p* = .058). However, the density of CC1 +ve oligodendrocytes (Bhat et al., 1996; Bin, Harris, & Kennedy, 2016; Kuhlmann, Remington, Maruschak, Owens, & Brück, 2007) was significantly decreased in infected animals (Figure 5g; *p* = .02). Similar trends were observed in the lumbar cord, although the differences were not significant (Figure 5h). Densities of microglia or astrocytes in white matter of cervical and lumbar cord were unchanged in ZIKV‐infected animals compared with mock‐infected controls (Figure 5g,h).

To determine if the vulnerability of oligodendrocytes reflects susceptibility to infection, we quantified the proportion ZIKV +ve cells that were CC1 positive. In spinal cord white matter at 4 dpi, 57.5% (±3.08 *SD*) of ZIKV +ve cells were also positive for CC1 (287 ZIKV +ve cells counted in cervical and lumbar cord sections from two independent animals). We next quantified the density of ZIKV +ve cells of each type at 5 dpi (white bars Figure 5e–h). Proportionally, the most to least susceptible cells were mature oligodendrocytes (CC1, ~15% ZIKV +ve), astrocytes (GFAP, ~10–13% ZIKV +ve), oligodendroglial lineage cells (OLIG2 ~8–11% ZIKV +ve), and neurons (NeuN, ~1% ZIKV +ve; Figure 4e–h). Microglia that were ZIKV positive (Iba1, ~23–27% ZIKV +ve; Figure 4g,h) might have been directly infected or have phagocytosed other infected cells.

In summary, in *Ifnar1* knockout mice, within the time frame examined, all neural cell types could be productively infected with ZIKV PE243, but only a small proportion of post‐mitotic neurons was targeted. Although the proportions of oligodendrocytes and astrocytes that were ZIKV +ve were similar, oligodendrocytes appeared most vulnerable, resulting in decreased densities in white matter.

### 3.6 Perinatal Zika virus infection causes mild neuroinflammation

**FIGURE 6:**
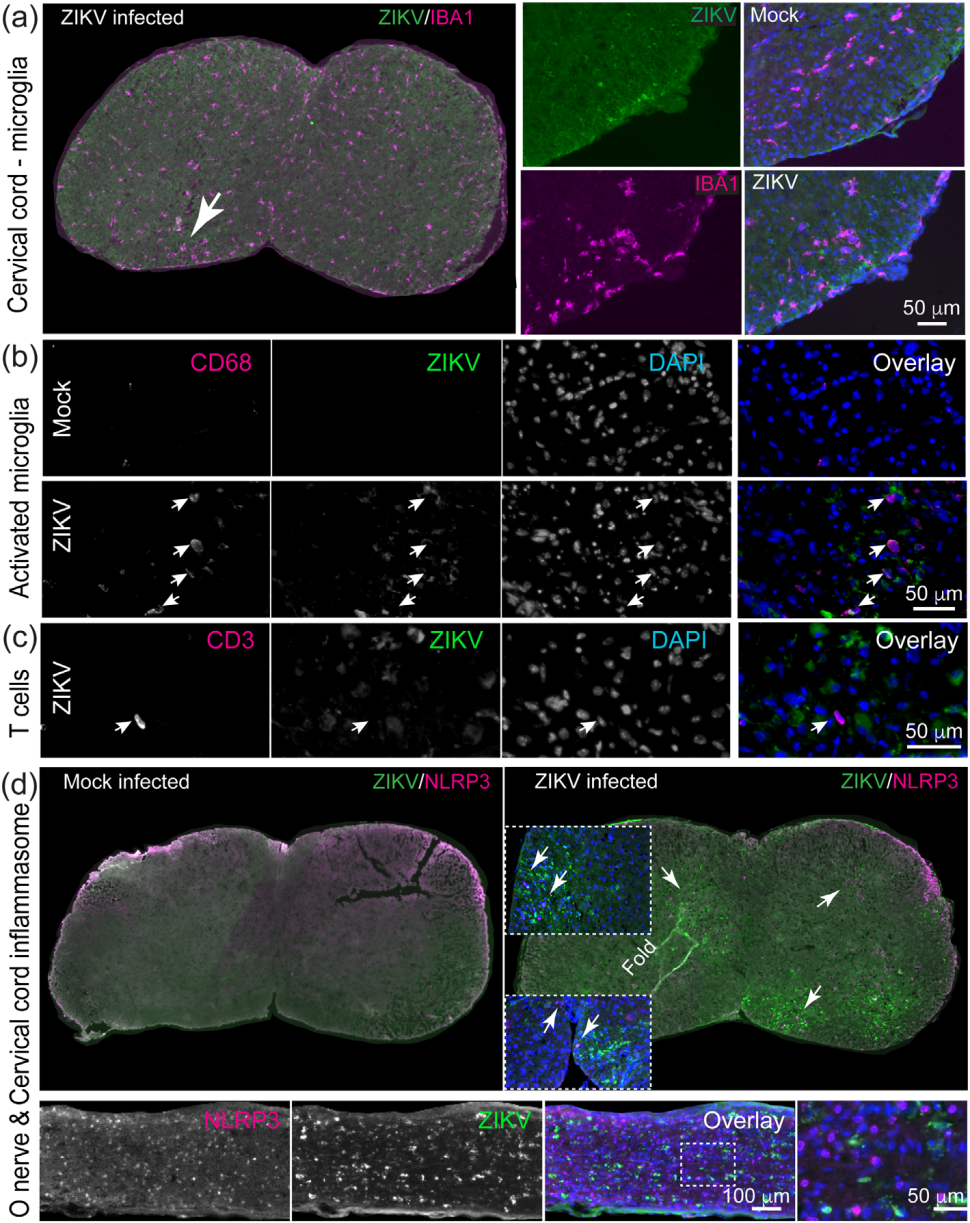
Mild neuroinflammation in ZIKV‐infected mice. (a) Representative immunofluorescence images of IBA1 and ZIKV in the cervical spinal cord of ZIKV‐infected mice and mock‐infected controls. The density of IBA1 +ve microglia is similar in both, however, IBA1 +ve cells were often observed at slightly increased densities at sights where ZIKV +ve cells were present (arrow in overview). (b) CD68 +ve “activated” microglia/macrophages are largely absent in mock‐infected controls (upper panel) and localized to areas containing ZIKV +ve cells in infected mice (lower panel). Images are of ventral (CD68) columns of lumbar spinal cord. (c) In contrast to our EAE control (not shown) only very rare CD3 +ve T lymphocytes were observed in the parenchyma of the spinal cord white matter of ZIKV‐infected mice. Image is of dorsal columns of the lumbar spinal cord. (d) NLRP3 +ve cells were absent in mock‐infected control tissue, but were observed, mainly but not exclusively, in regions of ZIKV +ve cells in spinal cord and optic nerve. Images are from mice at 4 dpi (a and cervical cord in d) or 5/6 dpi (b, c and optic nerve in d) [Color figure can be viewed at wileyonlinelibrary.com]

At the point of euthanasia, our mice showed no gross pathological changes that would explain the neurological signs. We therefore asked if these signs might reflect a neuroinflammatory process. Using antibody to IBA1, we demonstrated a propensity for microglia to cluster around ZIKV +ve cells (Figure 6a and Figure S2), however overall microglial densities in white matter were similar to control (Figures 5g,h and 6a). Using anti‐CD68 (a marker of activated microglia/macrophages) and anti‐CD3 (a T lymphocytes marker), we observed a localized activation of microglial/macrophages (Figure 6b), but only very rare T cells in the CNS parenchyma (Figure 6c). As the NLRP3 inflammasome was recently implicated in white matter injury in the perinatal and neonatal period in children (Holloway et al., 2021), we co‐stained spinal cord and optic nerve sections with anti‐NLRP3. We found NLRP3 +ve cells mainly in association with ZIKV +ve cells (Figure 6d). NLRP3 is likely expressed by microglia, however, as antibodies to NLRP3 and IBA1 are raised in the same species, we were unable to co‐stain. Comparing Figure 6a,d (right panel), the densities of IBA1 +ve cells and NLRP3 +ve cells in the proximity of ZIKV +ve cells, appear similar. We conclude that neurological signs might be the consequence of neuroinflammation. The reason for the rapid clinical course, culminating in death of the *Ifnar1* knockout mice, is unlikely to be related to CNS pathology.

## 4 DISCUSSION

ZIKV was first isolated in 1947; however, it was only during recent outbreaks in Micronesia (2007), French Polynesia (2013), and Brazil (2015) that it became clear that congenital infection with circulating Asian ZIKV is associated with a wide range of neuropathology and clinical signs and symptoms referred to as CZS (reviewed in Walker et al., 2019). The reason why some congenitally infected children appear asymptomatic at birth but subsequently experience transient or longer‐lasting postnatal neurodevelopmental delays (Familiar et al., 2020; Mulkey et al., 2020; Peçanha et al., 2020; Pimentel et al., 2021), is obscure. Here, using a mouse model of perinatal infection, we confirmed our earlier observations in cell culture (Cumberworth et al., 2017; Schultz et al., 2021), that postmitotic neurons are rather refractory to infection, whilst newly generated CNS glia are considerably more susceptible, leading to a reduction in the density of oligodendrocytes. Our data might help predict pathological changes in humans infected perinatally and explain the emergence of neurodevelopmental delays postnatally.

In our mouse model, we found most ZIKV +ve cells in the CNS were located in white matter, and the majority co‐labeled with antibody CC1, which labels Quaking (QKI) 7, an RNA‐binding protein highly upregulated in myelinating oligodendrocytes (Bin et al., 2016). We found significantly reduced densities of CC1 +ve cells in white matter and confirmed by electron microscopy that oligodendrocytes were dying. However, by immunostaining, only approximately 16% of cleaved caspase 3+ ve cells, were positive for CC1. We speculate this reflects that QKI7 is downregulated in apoptotic oligodendrocytes. Nonetheless, it is likely that some of the cleaved caspase 3 +ve/CC1 −ve cells are OPCs, although we were unable to confirm this as the fixation required to inactive ZIKV (8% paraformaldehyde) is not compatible with tissue staining with antibody to NG2, the classical OPC marker. Certainly, we previously showed in cell culture that OPCs are vulnerable to ZIKV infection (Figure 3b, Cumberworth et al., 2017). In the current study we showed that NLRP3 is expressed in proximity to ZIKV +ve cells. Recently, Holloway et al. (2021) showed that microglial activation of the NLRP3 inflammasome drives developmental hypomyelination through dysregulation of Activin A signaling, which promotes developmental myelination (Goebbels et al., 2017; Miron et al., 2013). Consequently, both cell death and impaired signaling are likely to impact developmental myelination following ZIKV infection.

Oligodendrocyte death is followed after some delay by loss of compact myelin (Pohl et al., 2011; Traka et al., 2010). However, the regenerative properties of CNS myelin are well known (Franklin & Ffrench‐Constant, 2017) and impaired developmental myelination can recover in both humans (Yan et al., 2019 and reviewed in Malik, Muthusamy, Mankad, Shroff, & Sudhakar, 2020), and animal models (Câmara et al., 2009; Yool et al., 2001). Consequently, if the vascular and neuronal “scaffoldings” are intact, and microglia downregulate the NLRP3 inflammasome, then ZIKV‐associated oligodendrocyte injury might represent a temporary pathology. Certainly, it is one that might be difficult to detect by clinical examination in new‐borns. Indeed, MRI observations in clinically unremarkable congenitally infected infants revealed high signal in T_2_ weighted images of the white matter (Brasil et al., 2016), potentially reflecting otherwise silent myelin changes. Further, in a study of infected mothers in Rio de Janeiro, 29% of pregnancies with third trimester infection had adverse outcomes including dysphagia, clonus, hyperreflexia, hypertonicity, and irritability (Table S2, Brasil et al., 2016), suggesting potential white matter involvement, as in the hypomyelinating leukodystrophies (Adang et al., 2017). Thus, myelin changes should not be ruled out in apparently asymptomatic new‐borns.

Earlier experimental studies also provided support for myelin involvement, including reports of ZIKV‐related disruption to OPC development and myelin deposition in mouse models of direct CNS ZIKV inoculation, embryonically (E15.5) or postnatally (P0; Zhang et al., 2017; Li et al., 2018). In contrast to the current study, these studies involved ZIKV inoculation at timepoints prior to the differentiation of OPCs into myelinating oligodendrocytes. Non‐human primate studies have also provided support for myelin involvement in the pathogenesis of ZIKV infection of the CNS. Oligodendroglial development was impaired in fetuses of mid‐gestation olive baboons infected systemically with a French Polynesian ZIKV isolate (H/PF/2013; Gurung et al., 2019) and white matter hypoplasia were reported in preterm pigtail macaque following systemic infection of the pregnant dam with a Cambodian isolate (FSS13025; Adams Waldorf et al., 2016). As in the current study, these non‐human primates were killed before the long‐term consequences were known and it will be important for future studies to address later outcomes, within the limitations of animal welfare issues.

ZIKV‐related myelin changes might not be limited developmental aspects. Acute myelitis and meningoencephalitis (Neri et al., 2018; Mécharles et al., 2016; Carteaux et al., 2016; Brito Ferreira et al., 2020), have also been described and may indicate transient focal demyelination due to ZIKV‐related oligodendroglial injury.

Whilst myelin can be restored following dysmyelination and/or demyelination (Franklin & ffrench‐Constant, 2017), it has been shown using an experimental mouse model of primary oligodendrocyte cell death, that demyelination and repair are followed months later by fatal secondary disease characterized by extensive myelin and axonal loss (Traka et al., 2016). These data demonstrate that primary oligodendrocyte death is sufficient to trigger an adaptive autoimmune response against myelin (Traka et al., 2016) and raise the concerning possibility that apparently minor consequences of ZIKV infection could lead later, in genetically susceptible individuals (International Multiple Sclerosis Genetics Consortium et al., 2011; Manet et al., 2020), to autoimmune‐mediated demyelination.

## 5 CONCLUSION

The relation between ZIKV infection in pregnancy and adverse neurological outcomes will become clearer when longitudinal studies are published (Wilder‐Smith et al., 2019). Our data highlight the need for lifetime monitoring in susceptible individuals, particularly in relation to development of autoimmune mediated demyelination. They may also help explain postnatal neurodevelopmental delays in children who appeared asymptomatic at birth; although alterations in other developmental processes in the perinatal and early postnatal period, aside from myelination, might also contribute (Kostović et al., 2019).

## CONFLICT OF INTEREST

The authors declare no conflicts of interest.

## AUTHOR CONTRIBUTIONS

**Verena Schultz, Jennifer A Barrie, Julia M. Edgar:** Conducted experiments and analysed data. **Claire L Donald:** Prepared and provided viral stocks. **Margaret Mullin, Julia M. Edgar, Colin L. Crawford:** Conducted electron microscopy. **Thomas J. Anderson:** Described clinical signs in mice. **Hugh J. Willison, Julia M. Edgar, Alain Kohl, Susan C. Barnett, Christopher Linington, Tom Solomon:** Obtained funding and contributed discussion. **Julia M. Edgar, Verena Schultz:** Designed study and wrote the article, which was edited and agreed by all authors.

## Supporting information


**Figure S1** Fluorescence micrographs of sagittal section of cerebellum and longitudinal sections of cervical and lumbar spinal cord show that ZIKV +ve cells tend to occur in cluster, largely in the MBP +ve white matter. In a and b, sections from mock‐infected and ZIKV‐infected animals are shown. In c, low and high magnification views of an area of interest in an infected animal are shown.
**Figure S2**. Fluorescence micrographs of spinal cord show that focally increased densities of IBA1 +ve microglia/macrophages tend to occur at sites of ZIKV +ve cells, despite that overall IBA1 +ve densities are not increased.

**Table S1** Supporting Information

## Data Availability

The data that support the findings of this study are available from the corresponding author upon reasonable request.
