# Supplementary material for: Oligodendrocytes are susceptible to Zika virus infection in a mouse model of perinatal exposure: Implications for CNS complications
Source: Glia. 2021 May 4;69(8):2023–36. doi: 10.1002/glia.24010 (PMC9216243; doi:10.1002/glia.24010)
Supplement: Supplementary file 1 — Figure S1 Fluorescence micrographs of sagittal section of cerebellum and longitudinal sections of cervical and lumbar spinal cord show that ZIKV +ve cells tend to occur in cluster, largely in the MBP +ve white matter. In a and b, sections from mock‐infected and ZIKV‐infected animals are shown. In c, low and high magnification views of an area of interest in an infected animal are shown. Figure S2. Fluorescence micrographs of spinal cord show that focally increased densities of IBA1 +ve microglia/macrophages tend to occur at sites of ZIKV +ve cells, despite that overall IBA1 +ve densities are not increased. [file GLIA-69-2023-s002.docx]

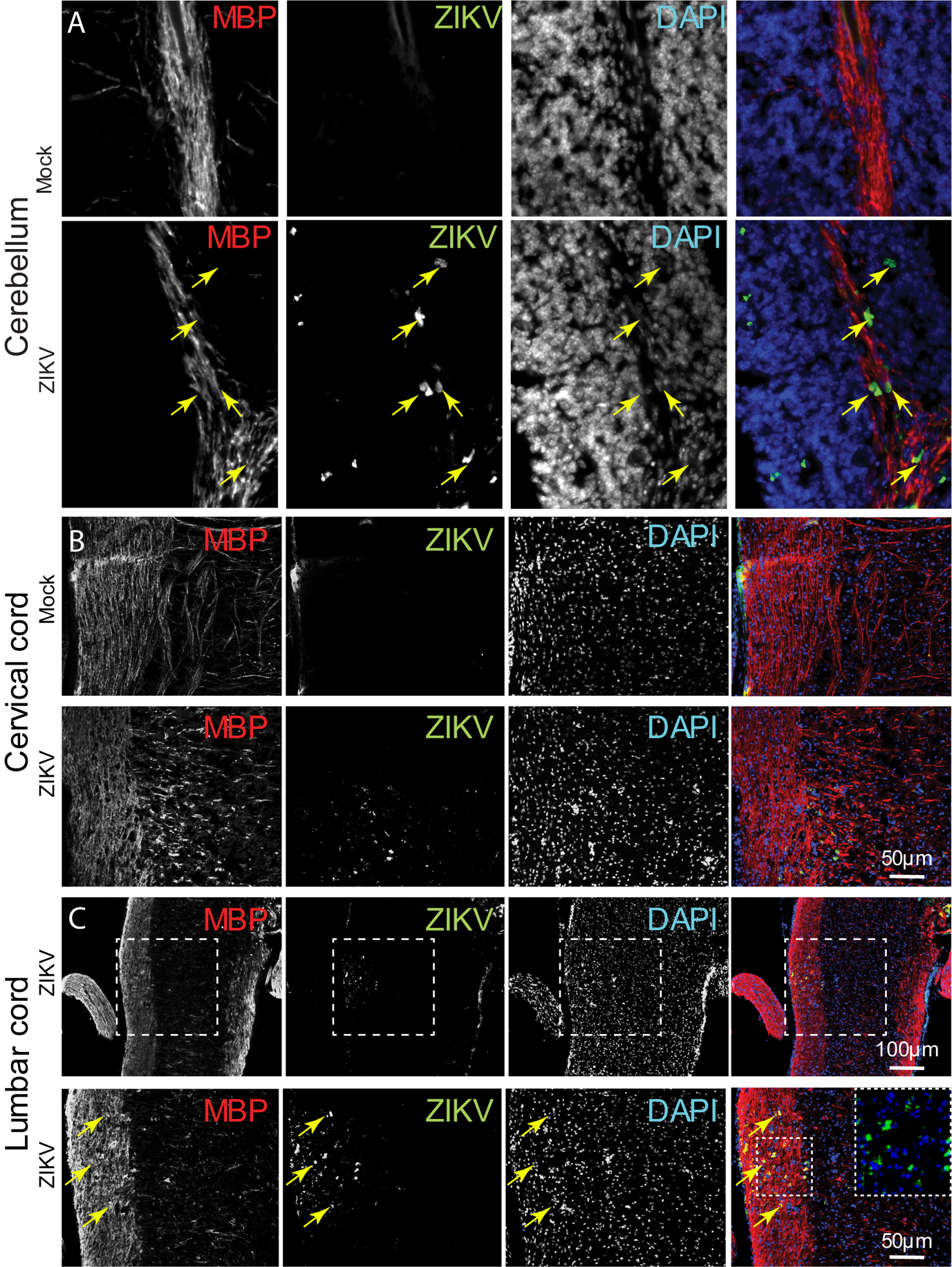
Supplementary Figure 1. Fluorescence micrographs of sagittal section of cerebellum and longitudinal sections of cervical and lumbar spinal cord show that ZIKV +ve cells tend to occur in cluster, largely in the MBP +ve white matter. In a and b, sections from mock infected and ZIKV infected animals are shown. In c, low and high magnification views of an area of interest in an infected animal are shown.


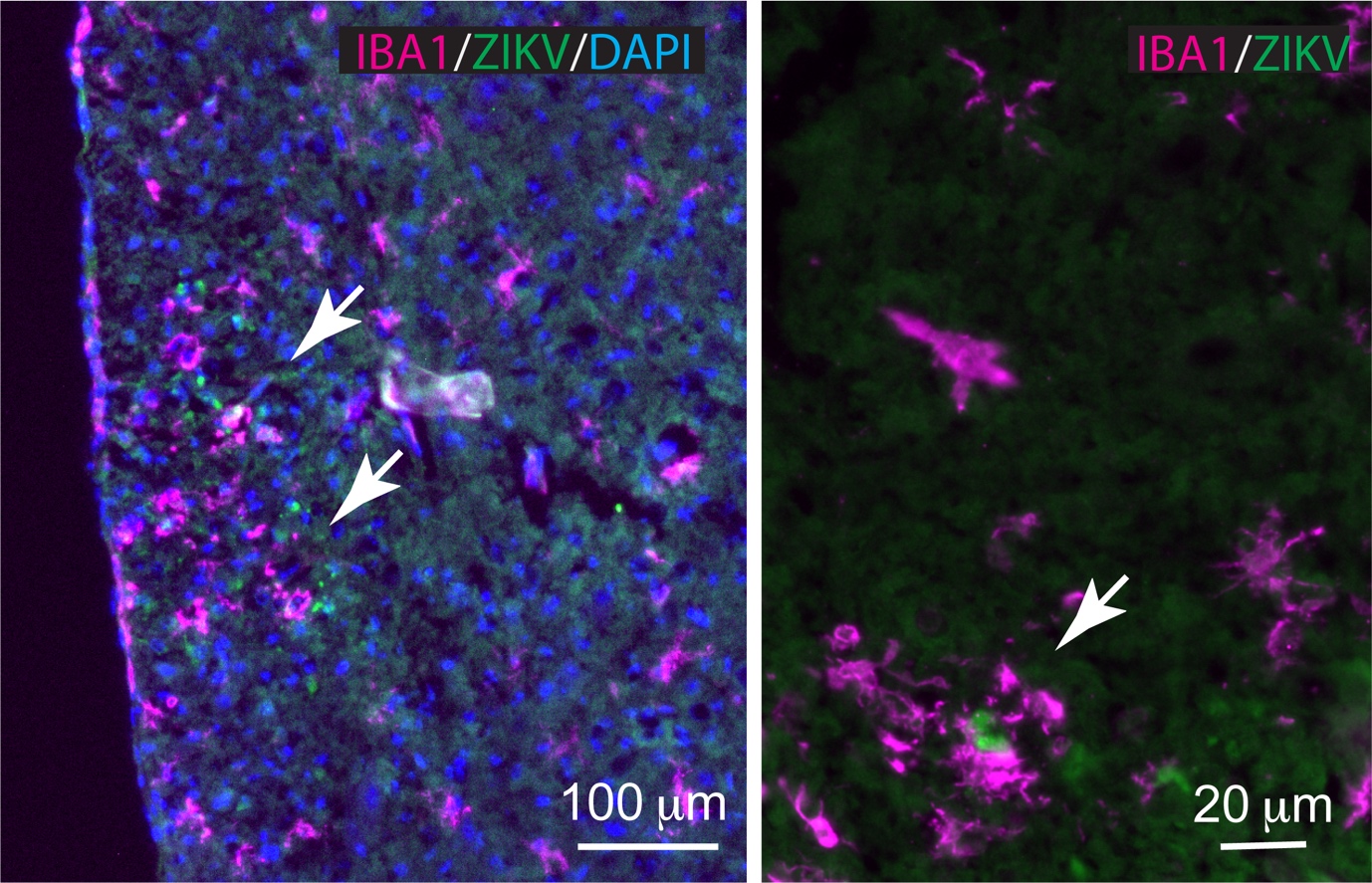


Supplementary Figure 2. Fluorescence micrographs of spinal cord show that focally increased densities of IBA1 +ve microglia/macrophages tend to occur at sites of ZIKV +ve cells, despite that overall IBA1 +ve densities are not increased.
