# Supplementary material for: Oligodendrocytes are susceptible to Zika virus infection in a mouse model of perinatal exposure: Implications for CNS complications
Source: Glia. 2021 May 4;69(8):2023–36. doi: 10.1002/glia.24010 (PMC9216243; doi:10.1002/glia.24010)
Supplement: Supplementary file 2 — Table S1 Supporting Information [file GLIA-69-2023-s001.docx]

| **Litter #** | **Date of procedure** | **Genotype** | **DOB** | **No. of pups** | **Age infected** | **Viral  stock** | **PFU/ animal** | **Age at death/culling** | **Status on day of culling/fixation** | **Exp #** |
| --- | --- | --- | --- | --- | --- | --- | --- | --- | --- | --- |
|  |  |  |  |  |  |  |  |  |  |  |
| **ZP1** | 29.05.2018 | Ifnar1 ko | 24.05.2018 | 5 | P5 | mock (N'Pro medium) | 0 | P10 | Appeared normal | ZP1.5 |
|  |  |  |  |  | P5 | PE243 (N'Pro) | 7.5*10^5 | P10 | Pup was deteriorated, dragging hindlimbs or immobile, failed to react when lifted, lying outside of nest, cold to touch | ZP1.1 |
|  |  |  |  |  | P5 | PE243 (N'Pro) | 7.5*10^5 | P10 | Pups showed neurological signs (Fig 1 and video) including dragging of hind limb, or failure to right | ZP1.2 |
|  |  |  |  |  | P5 | PE243 (N'Pro) | 7.5*10^4 | P10 |  | ZP1.3 |
|  |  |  |  |  | P5 | PE243 (N'Pro) | 7.5*10^4 | P10 |  | ZP1.4 |
| **ZP2** | 15.07.2018 | Ifnar1 ko | 09.07.2018 | 3 | P6 | mock (N'Pro medium) | 0 | P12 | Appeared normal | ZP2.6 |
|  |  |  |  |  | P6 | PE243 (N'Pro) | 7.5*10^4 | P12 | Found dead (FD; previous day, very mild weakness in right front paw). | ZP2.4 |
|  |  |  |  |  | P6 | MR766 (VeroE6) | 7.5*10^4 | P11 | unresponsive, not moving, slow heartbeat | ZP2.3 |
|  | 15.07.2018 | WT | 09.07.2018 |  | P6 | PE243 (N'Pro) | 7.5*10^5 | P15 | slow movement, but no weakness in limbs, slightly shakey, hunched posture | ZP2.8 |
|  |  |  |  |  | P6 | PE243 (N'Pro) | 7.5*10^4 | P25 | Appeared normal | ZP2.11 |
|  |  |  |  |  | P6 | MR766 (VeroE6) | 7.5*10^5 | P17 | Appeared normal | ZP2.9 |
|  |  |  |  |  | P6 | MR766 (VeroE6) | 7.5*10^4 | P25 | Appeared normal | ZP2.12 |
|  |  |  |  |  | P6 | mock (N'Pro medium) | 0 | P25 | Appeared normal | ZP2.13 |
|  | 15.07.2018 | WT | 10.07.2018 |  | P5 | PE243 (N'Pro) | 7.5*10^5 | P16 | Appeared normal | ZP2.10 |
|  |  |  |  |  | P5 | PE243 (N'Pro) | 7.5*10^4 | P24 | Appeared normal | ZP2.14 |
|  |  |  |  |  | P5 | MR766 (VeroE6) | 7.5*10^5 | P11 | Piloerection, no movement of the right hindlimb, shakey when moving | ZP2.5 |
|  |  |  |  |  | P5 | MR766 (VeroE6) | 7.5*10^4 | P12 | Piloerction and hunched posture | ZP2.7 |
|  |  |  |  |  | P5 | mock (N'Pro medium) | 0 | P24 | Appeared normal | ZP2.15 |
| **ZP3** | 23.07.2018 | Ifnar1 ko | 18.07.2018 | 7 | P5 | mock (N'Pro medium) | 0 | P11 | Appeared normal | ZP3.5 |
|  |  |  |  |  | P5 | PE243 (N'Pro) | 8*10^4 | P11 | Very weak, limited movement, struggling to right itself | ZP3.4 |
|  |  |  |  |  | P5 | PE243 (N'Pro) | 8*10^4 | P11 | FD, died recently, no rigor mortis | Tissue unusable |
|  |  |  |  |  | P5 | PE243 (C636) | 8*10^4 | P10 | Appeared lethargic and dehydrated | ZP3.3 mosquito propagated virus |
|  |  |  |  |  | P5 | PE243 (C636) | 8*10^4 | N/A | Liver was injured during ip injection. | N/A |
|  |  |  |  |  | P5 | MR766 (N'Pro) | 8*10^4 | P9 | FD | ZP3.1 |
|  |  |  |  |  | P5 | MR766 (N'Pro) | 8*10^4 | P9 | Appeared sick, culled | ZP3.2 |
| **ZP4** | 10.10.20 | Ifnar1 ko | 5.10.20 | 8 | P5 | mock (N'Pro medium) | 0 | P10 | Appeared normal | ZP4.1 |
|  |  |  |  |  | P5 | mock (N'Pro medium) | 0 | P10 | Appeared normal | ZP4.2 |
|  |  |  |  |  | P5 | mock (N'Pro medium) | 0 | P10 | Appeared normal | ZP4.3 |
|  |  |  |  |  | P5 | PE243 (N'Pro) | 7.5*10^4 | P10 | FD full stomach (milk) | ZP4.4 |
|  |  |  |  |  | P5 | PE243 (N'Pro) | 7.5*10^4 | P10 | FD empty stomach (no milk) | ZP4.5 |
|  |  |  |  |  | P5 | PE243 (N'Pro) | 7.5*10^4 | P10 | FD partially cannibalised | ZP4.6 |
|  |  |  |  |  | P5 | PE243 (N'Pro) | 7.5*10^4 | P10 | FD full stomach, no sciatic nerve | ZP4.7 |
|  |  |  |  |  | P5 | PE243 (N'Pro) | 7.5*10^4 | P10 | Appeared slightly small, full stomach | ZP4.8 |
| **ZP5** | 26.10.2020 | Ifnar1 ko | 21.10.2020 | 6 | P5 | mock (N'Pro medium) | 0 | P9 | Appeared normal | ZP5.3 |
|  |  |  |  |  | P5 | mock (N'Pro medium) | 0 | P9 | Appeared normal | ZP5.5 |
|  |  |  |  |  | P5 | mock (N'Pro medium) | 0 | P9 | Appeared normal | ZP5.6 |
|  |  |  |  |  | P5 | PE243 (N'Pro) | 7.5*10^4 | P9 | Appeared slightly small, full stomach | ZP5.4 |
|  |  |  |  |  | P5 | PE243 (N'Pro) | 7.5*10^4 | P9 | Appeared slightly small, full stomach | ZP5.1 |
|  |  |  |  |  | P5 | PE243 (N'Pro) | 7.5*10^4 | P9 | Appeared slightly small, full stomach | ZP5.2 |
| **ZP6** | 26.10.2020 | Ifnar1 ko | 21.10.2020 | 7 | P5 | mock (N'Pro medium) | 0 | P9 | Appeared normal | ZP6.1 |
|  |  |  |  |  | P5 | mock (N'Pro medium) | 0 | P9 | Appeared normal | ZP6.2 |
|  |  |  |  |  | P5 | mock (N'Pro medium) | 0 | P9 | Appeared normal | ZP6.3 |
|  |  |  |  |  | P5 | PE243 (N'Pro) | 7.5*10^4 | P9 | Appeared slightly small, full stomach | ZP6.4 |
|  |  |  |  |  | P5 | PE243 (N'Pro) | 7.5*10^4 | P9 | Appeared slightly small, full stomach | ZP6.5 |
|  |  |  |  |  | P5 | PE243 (N'Pro) | 7.5*10^4 | P9 | Appeared slightly small, full stomach | ZP6.6 |
|  |  |  |  |  | P5 | PE243 (N'Pro) | 7.5*10^4 | P9 | Appeared slightly small, full stomach | ZP6.7 |
